# A Case Study of Ergonomic Risk Assessment in Slovakia with Respect to EU Standard

**DOI:** 10.3390/ijerph21060666

**Published:** 2024-05-23

**Authors:** Daniela Onofrejova, Miriam Andrejiova, Denisa Porubcanova, Hana Pacaiova, Lydia Sobotova

**Affiliations:** 1 Faculty of Mechanical Engineering, Department of Safety and Production Quality, Technical University of Kosice, Letna 1/9, 04200 Kosice-Sever, Slovakia; denisa.porubcanova@tuke.sk (D.P.); hana.pacaiova@tuke.sk (H.P.); 2 Faculty of Mechanical Engineering, Department of Applied Mathematics and Computer Science, Technical University of Kosice, Letna 1/9, 04200 Kosice-Sever, Slovakia; miriam.andrejiova@tuke.sk; 3 Faculty of Mechanical Engineering, Department of Business Management and Environmental Engineering, Technical University of Kosice, Letna 1/9, 04200 Kosice-Sever, Slovakia; lydia.sobotova@tuke.sk

**Keywords:** prevention of musculoskeletal disorders, ergonomic risk, legislation, ergonomic assessment methods

## Abstract

Attention on work-related musculoskeletal disorders (WMSDs) involves statistical surveys showing an increasing trend in the incidence of WMSDs. Technological development has led to new tools and methods for the assessment of physical load at work. These methods are mostly based on the direct sensing of appropriate parameters, which allows more precise quantification. The aim of this paper is to compare several commonly used methods in Slovakia for the assessment of ergonomic risk reflecting current EU and Slovak legislative regulations. A Captiv wireless sensory system was used at a car headlight quality control assembly workplace for sensing, data acquisition and data processing. During the evaluation of postures and movements at work, we discovered differences in the applicable standards: Decree 542/2007 Coll. (Slovak Legislation), the STN EN 1005-4+A1, and the French standards default in the Captiv system. Standards define the thresholds for hazardous postures with significant differences in several evaluated body segments, which affects the final evaluation of the measurements. Our experience from applying improved risk assessment methodology may have an impact on Slovak industrial workplaces. It was confirmed that there is a need to create uniform standards for the ergonomic risk assessment of body posture, including a detailed description of the threshold values for individual body segments.

## 1. Introduction

### 1.1. Musculoskeletal Disorders and Their Possible Reduction

Health and wellbeing are major societal priorities. According to surveys conducted by the European Agency for Occupational Safety and Health at Work (EU OSHA), approximately three out of five workers complain of MSDs (musculoskeletal disorders) [1]. Statistics on the incidence of occupational diseases in Slovakia over the last 20 years show a variable trend. However, the latest statistics from 2021, published by the National Centre for Health Information [2], show an increasing trend in the occurrence of WMSDs (work-related musculoskeletal disorders); therefore, attention should be paid to their reduction. The overall increase in the number of occupational diseases, compared to 2020, was 169 cases, with a total of 423 cases of occupational diseases occurring in 2021, with the most affected workers being in motor vehicle manufacturing, metal structures excluding machinery and equipment, and healthcare [2]. The most common WMSDs include pain in the neck, upper limbs, and lower back [3]. Pain in the lower limbs seems to be less frequently claimed by workers, which is probably due to their natural adaptation to the load, as their primary function is walking and standing, during which they bear the weight of their body [4]. Our research goal is to concentrate on the upper body segments following our plan to test exoskeletons supporting that body part.

The aging of the population raises many issues and provides many opportunities. It intensifies the requirement for long-term care, healthcare, and a better-skilled workforce, and increases the demand for age-friendly environments. The most prominent studies suggest policies and practices that support life-long learning, a workforce that comprises both younger and older workers, and gradual retirement [5]. Most of the recorded cases of occupational diseases occurred in the age group of 50–59 years, i.e., in employees with a high probability of tissue wear and tear due to long-term loading, which again points to the necessity of preventing such diseases by the proper limitation of workload and work movements and positions and the application of ergonomic principles in practice [2,6]. 

Ergonomics design focuses on the actual activity of operators. The methodology described in the European Standard EN 16710-2 [7] therefore increases the effectiveness and efficiency of the machinery or system being designed; improves human working conditions; and reduces adverse effects on health, safety, and performance. Applying this will raise productivity; improve work quality; reduce technical support, maintenance, and training needs; and enhance user/operator satisfaction. The risk assessment model in the EN ISO 11228-1 document [8] allows the estimation of the risk associated with a manual material handling task. It takes into consideration the hazards (unfavorable conditions) related to manual handling tasks and the time spent performing them. The European Standard STN EN 1005-4+A1 [9] ”Machine safety. Human physical performance. Part 4: Assessment of working postures and movements when working on machinery.“ provides guidance on the assessment of potential health risks associated with work only in working positions and during movements of machinery, i.e., during its assembly, installation, operation, adjustment, maintenance, cleaning, repair, transport, and dismantling, which must be taken into account when designing machinery or its components. This European Standard specifies the requirements for positions and movements in which there is no or minimal external force. These requirements are intended to reduce health risks for almost all healthy adult workers. Similarly, with the Industry 4.0 adaptation, workplace design approaches require risk assessments that emphasize the interactions between the operator, the robot, and the work environment. Guidelines for the design of safe human–robot collaborative assembly are being developed and classified [10].

The assessment of physical exposure in general should include three dimensions of a load: level (amplitude), repetition, and duration [3]. Available methods usually focus on the assessment of the applied load in different working postures, while other factors such as repetition and duration of postures or muscle strength are less frequently considered [11]. In practice, a number of methods are used to assess workload, which are selected according to the nature of the activity being performed and the relevant body part. The main types of used methods are categorized into three groups: (1) self-reported by workers; (2) observational methods: both simple techniques developed for systematically recording workplace exposure, and advanced techniques for the assessment of postural variation during highly dynamic activities; (3) real-time measurements using monitoring instruments with sensors attached directly to the subject measuring the exposure variables at work [12]. We describe the most used methods in the following part. 

In general, REBA (Rapid Entire Body Assessment) and RULA (Rapid Upper Limb Assessment) methods are often used for risk assessment in Slovakia, and other countries as well [13,14], as they are relatively easy and fast, and do not stress the workers in any way in the work cycle and movement habits associated with their work. Many of these methods have been designed based on long-term research, others have been built on the laws of biomechanics and ergonomics, and some have been developed by modifying already existing methods to obtain better sensitivity of the assessment methodology or to adapt the methodology to a specific type of work (e.g., working with loads) [12,15,16].

The REBA method is mainly used for the analysis of forced postures and postures of the upper limbs (arm, forearm, wrist), trunk, neck, and lower extremities. It distinguishes a type of grip and muscle activity. It identifies five levels of risk [17]. The RULA [18] is able to do more as it considers whether a working process has static or dynamic movement sequences [19]. The OWAS (Ovako Working posture Analysis System method) was intended to identify the frequency and time spent in the postures adopted in each task, study and evaluate the situation, and thus, recommend corrective actions. The OWAS identifies the most habitual back postures in workers (four postures), arms (three postures), legs (seven postures), and weight of the load handled (three categories) [20]. Other tools used to assess work postures and the resulting load on the body are the LUBA (Postural Loading on the Upper Body Assessment) system [21] and the ULRA (Upper Limb Risk Assessment) [22]. NIOSH (National Institute for Occupational Safety & Health) methodology is used for assessing lifting actions by means of a quantitative method based on intensity, duration, frequency, and other geometrical characteristics of lifting. Authors [23,24] have even explored the implementation of machine learning (ML) to classify biomechanical risk using the revised NIOSH lifting equation and signals from wearable sensors for acceleration and angular velocity. The OCRA (Occupational repetitive action) is a commonly applied method of evaluating the musculoskeletal load of the upper limbs caused by repetitive tasks and the risk of developing MSDs [25]. OCRA and ULRA assess the upper limb load based on body posture, exerted forces, and time. However, these methods primarily differ in how they identify variables that describe posture, forces, and time sequences. The authors of [26] analyzed the convergence of the OCRA and ULRA methods by comparing exposure and the assessed risk of developing MSDs at 18 repetitive task workstations. The results revealed that using different methods not only produces differences in exposure assessment but also differences in assignment to risk zones. Thus, evaluators should not rely on the output of a single risk assessment only. A method of load assessment, which is similar to the NIOSH lifting equation, is the STRAIN Index (SI) [27]. The values corresponding to the parameters as well as the resulting rating scale were developed based on research and biomechanical knowledge. Research conducted in the years 2000 and 2002 confirmed the high accuracy of this tool in assessing the riskiness of work positions. The authors of [28] focused on developing an inertial sensor-based approach to evaluate posture in industrial contexts, particularly in automotive assembly lines. 

There are many observational methods for evaluating working postures. One advantage is that those methods have a high degree of subjectivity. Motion capture kinematic suits and motion sensors applied on the body can ensure the objectivity of the assessment [29,30,31]. 

In collaboration with the INRS (French National Institute for Research and Safety), the TEA FRANCE, Vandoeuvre-lès-Nancy, has developed Captiv. The system is able to synchronize video sequences together with visual observations and wearable sensor data. The related software evaluates ergonomic risk with respect to physical load threshold values related to working positions and other physiological parameters as well [32]. We used that system for our research because it is a well-known European measurement synchronization platform for human behavior analysis equipped with wearable wireless sensors, a video capturing system, and visualization software using avatars. Wearable sensors enable us to collect all important motion and relevant ergonomic parameters, allow the real-time monitoring of workers’ postures, and have proven to be an asset for ergonomic studies due to their high accuracy [33]. The aim of our research was to compare results for the assessment of ergonomic risks by three different standards—the Slovak legislative regulation, the French (Captiv system) standard, and the EU ergonomic standard. Measurements were provided at a car headlight quality control assembly workplace. 

### 1.2. Important Issues of Standards for Physical Load Assessment Applied in Slovakia

In terms of standardization requirements (standards, laws), there is a valid legislation Decree 542/2007 Coll. in Slovakia [34,35] (hereafter referred to as SK Legislation) with details of the protection of health against physical loads at work and psychological and sensory workloads. Recently, STN EN 1005-4+A1 [9] (later referred to as STN EN) was also added to the EU standard translation. Both standards assess the physical load at work based on the working postures of individual body segments. However, when comparing them with each other, we concluded that many of the limiting boundaries of the postures in these standards do not coincide, which in practice means that the final evaluation of the workload may differ based on the standard used.

Our introductory research analysis discovered some differences in the above-mentioned standards. Table 1 shows that these standards differ in several parameters and, subsequently, in the overall system of assessment for the individual segments. In some parameters, the variation is moderate, but for some, the difference in the threshold values for individual postures is significant. It is evident in the case of the upper and lower extremities. In order to limit the movements of the upper and lower limbs, SK legislation in contrast with STN EN standard uses different parts of the limbs. The STN EN standard even specifies their movements with the exact limitation of the movement of the lower limb. This ultimately impacts on the final evaluation of the working postures, so we consider it appropriate to modify these thresholds in the currently used standards. 

Significant variations occur in the limits of acceptable movements for the head and neck region, where the SK Legislation refers to forward head flexion and backward head flexion, whereas the STN EN describes only forward head flexion, but with a considerably bigger range. Similarly, the zero axis, from which the limit values of movements are derived, does not completely coincide with this [9,32,35].

In the case of determining the limiting movements and postures for the trunk area during static work, the STN EN and the SK Legislation agree on limiting the bending to 60°. For dynamic work, the range of unacceptable postures is no longer the same, with a difference of up to 40° in the limitation of bending, with a bigger range permitted by the STN EN standard. For other postures and movements of the trunk at work, the STN EN and the Slovak Legislation agree on several parameters in the area of conditionally acceptable movements; namely, they allow bending with support in static work, and in the performance of dynamic work, they also allow bending with a frequency of less than 2/min, as well as bending of 60° with the same frequency [9,32,35].

If we want to proceed with the analysis and comparison between the SK Legislation and the STN EN, a rate of non-compliance is observed. In fact, for the delimitation of the movements of the upper and lower limbs, the SK Legislation and the STN EN use different parts of the limbs for the representation of their movements, and the STN EN does not even contain a chapter with a precise specification of the movements of the lower limbs. For a description of the limits and movements of the other parts of the body, according to both the SK Legislation and the STN EN, a general description applies, which excludes postures close to the limits of the ranges of movement and uncomfortable postures [9,32,35].

It is also important to note that, within the standards, a limb is often represented as a whole part, not specifically by single joints; for example, the upper limb is represented by the upper arm. To illustrate this with an example, Decree 542/2007 Coll. uses two points to define the position of the upper limb, namely the outer edge of the clavicle and the elbow joint. However, we do not consider this assessment to be sufficient, as this principle does not consider movements in the elbow joint or in the wrist, which has been a problematic part of the body in recent years, due to the trend of a high percentage of carpal tunnel. 

This raises the question of the adequacy of the SK Legislation and the STN EN in this respect, since each limb also contains articulations, the movements of which are more directive and more accurately measurable. It is therefore inadequate in practice to use such a representation of a limb by one part of it. The upper limb itself consists of the shoulder, elbow, and wrist joints, and likewise, the lower limb consists of articulations such as the hip, knee, and ankle. The movements of the individual joints cannot be represented by the movement of the whole part of the limb, which consequently makes such an assessment inappropriate. 

The essence of ergonomic risk assessment is to identify risk factors in the work environment that have the potential to cause damage to the musculoskeletal system, which we aim to minimize as part of the protection of human health. If the input parameters for the assessment are set correctly and the correct work procedure is followed using the chosen method, there is a high probability that the resulting assessment will yield the correct conclusions. 

Therefore, we focused on the evaluation of the deviations obtained by evaluating the same parameters measured with the Captiv system [32] during our research, according to the SK Legislation [34] and the STN EN [9]. The Captiv itself has pre-set threshold values [32,36] for acceptable, conditionally acceptable, and unacceptable postures, but these are based on the INRIS French standards, which also differ from the standards valid in the Slovak Republic. The inter-comparison of these standards is summarized in Table 1.

## 2. Materials and Methods

### 2.1. Captiv Measurement System

As we preferred new methods for the measurement of workers’ ergonomic parameters in our research, we started to use the Captiv wearable multisensory system that enables the measurement of human motion and provides post-processing in the form of multi-functional posture analysis, including applied load, musculoskeletal constraints, and frequency of repetitive movements and vibrations. The definition of postures in Captiv is based on the INRIS French standard. It offers flexible and scalable measurements, as well as an effective toolset for analyzing ergonomics in the workplace, occupational safety evaluation, HMI (Human–Machine Interface) prototyping, and even research, VR (virtual reality), and other applications [32,33,35]. 

The essence of ergonomic risk assessment is to identify risk factors in the work environment that have the potential to cause damage to the musculoskeletal system, which we aim to minimize as part of the protection of human health. If the input parameters for the assessment are set correctly and the correct work procedure is followed using the chosen method, there is a high probability that the resulting assessment will yield the correct conclusions. 

### 2.2. Measurement Procedure

#### 2.2.1. Measurement Plan

The basic principle of the ergonomic analysis in this article is to monitor the time that individual body segments (N—neck; RS—right shoulder; LS—left shoulder; RE—right elbow; LE—left elbow; UB—upper back; LB—lower back) spent in a specific working posture expressed in percentages. As mentioned above, we applied three different methods (standards: Captiv, STN EN, and SK Legislation).

The aims of the experimental plan and obtained data evaluation were the following:

▪ To measure the time individual body segments spend in a specific working posture for 5 workers using three assessment methods;
▪ To determine the time individual body segments spend in acceptable (Green), conditionally acceptable (Orange), and unacceptable (Red) working postures;
▪ To determine the difference between three methods: the SK Legislation (L), the STN EN (S), and the Captiv system (C).

The measurement plan consisted of the 5 following steps:

1. Calibration of Captiv sensors.
  Calibration is a very important step. All wireless sensors must be synchronized with the receiver unit. It is crucial to avoid magnetic disturbances.
2. Positioning of the sensors on the worker.
  The position may differ based on the activity to be performed and the observed location of the expected strain.
3. Processing the measured values of time for each body segment and evaluating the risk.
  An algorithm evaluates the proportion of time for each evaluated body segment remaining in an acceptable, conditionally acceptable, or unacceptable position. Such an evaluation is provided separately for each of the three different standards.
4. Evaluation of the measured values and their comparison.
  The results of the ergonomic risk analysis for each of the 3 different standards were compared, and the differences between them were calculated by statistical tests.
5. Description of the findings from the processed measured and compared values.
  This step was to identify if there were differences in the proportion of time spent by a body segment in acceptable, conditionally acceptable, and unacceptable risk.

#### 2.2.2. Description of Experimental Workplace

Five workers aged 23 to 45 years participated in the measurement. They provided their working tasks while the Captiv system captured their movements in real time. The workplace was situated at an assembly station. Measurements were made for each of the 5 workers three times to obtain more reliable results for the statistical evaluation. During the work activity, the worker performed the following work operations: took the label from the printer and stuck it on the HDL; removed the HDL from the pallet and put it on the Xpert table; checked the light according to the test instructions; and put the OK light in the box and sent it to the Q-gate (the NIO part was put away in the red box). Work was conducted in a standing position. The nature of the work was rather static, with a semi-dynamic component of movement when picking and putting away the part. The microclimatic conditions at the time of measurement were as follows: atmospheric pressure 1012 hPa, air temperature 22 °C, humidity 53%.

#### 2.2.3. Description of Sensors and Their Placement

**Figure 1:**
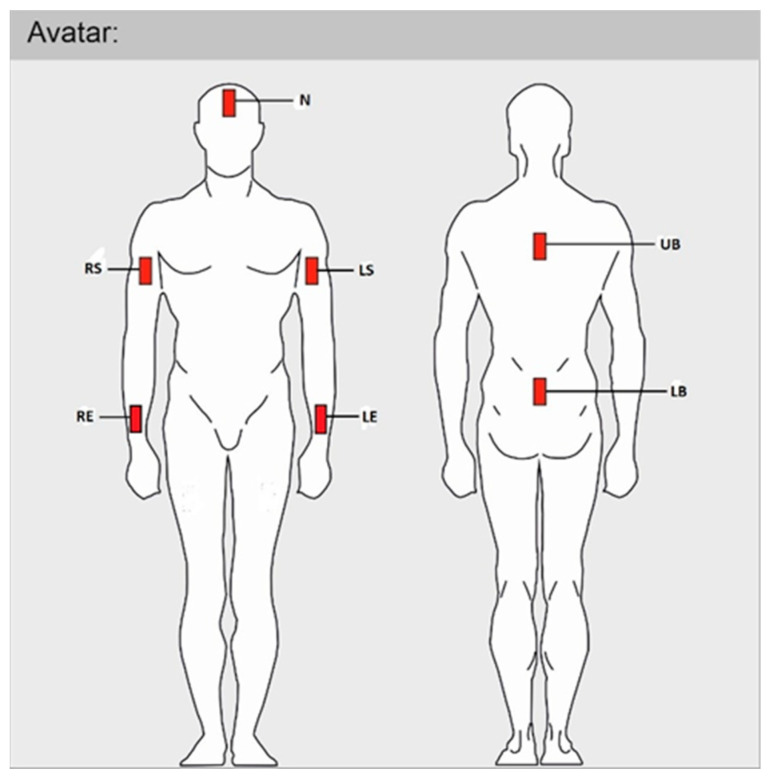
Sensor placement on avatar in Captiv: N—neck; RS—right shoulder; LS—left shoulder; RE—right elbow; LE—left elbow; UB—upper back; LB—lower back.

The measured joints, according to the standards, were the neck, back (axis defined as the line of the pelvis–vertebral segment T2), left and right shoulders, and left and right forearms. Three-dimensional visualization was in the form of an avatar; the workers were equipped with wearable wireless sensors on their back (for upper body tracking) and on the lumbar region of the sacrum (for lower back and hip tracking). To measure the physical load for the ergonomic risk assessment of work postures, 7 motion IMU sensors were used in relation to the frequency and length of work movements. The sensor placement (Figure 1) on the avatar in Captiv was chosen according to the following measured joints for results in upper body parts: N—neck; RS—right shoulder; LS—left shoulder; RE—right elbow; LE—left elbow; UB—upper back; LB—lower back. The sensor located in the UB provides information about the position of the neck. Measured body segments and their labels are explained in Table 2.

The thresholds were determined by three standards, the SK Legislation, STN EN, and INRIS standards, built into the Captiv system, which took part in the evaluation and sorting of the collected data according to the time spent in each position.

### 2.3. Statistical Testing for the Evaluation of Measured and Processed Data

Basic methods were used to analyze the measured data on the percentage of time each body segment remained in the acceptable, conditionally acceptable, and unacceptable positions: descriptive statistics and statistical hypothesis testing methods. 

The statistical hypothesis was based on the decision rule: to reject or accept the null hypothesis using the *p*-value. If the *p*-value is less than the specified significance level α, then the null hypothesis is rejected. If the *p*-value is equal to or bigger than the chosen significance level α, then the null hypothesis is not rejected.

The resulting values were tested by the paired *t*-test. The condition of normality is a prerequisite for its use. In practice, two main tools are used to assess normality: graphical representation of data and visual assessment of normality (e.g., histogram, Q-Q plot, P-P plot), or testing using statistical tests (e.g., Shapiro–Wilk normality test, Kolmogorov–Smirnov test, Anderson–Darling test, etc.) [37]. Normality assessment was provided by the Shapiro–Wilk normality test, which is the most frequently used for normality testing in the case of small to medium ranges of data up to 2000. The null hypothesis was “the sample distribution is normally distributed”, and the alternative hypothesis was “the sample distribution is not normally distributed”. We used the statistical software R (an official part of the Free Software Foundation’s GNU project, which can be downloaded as open-source software).

For the evaluation, we used the differentiation of the values of the measured percentages from the different stressed body parts by means of three different evaluation methods. We calculate the difference in each working position (acceptable, conditionally acceptable, unacceptable) at each measurement point for each worker according to the following formula:

(1) $${∆}_{k1,r}=Z_{Captiv,k,r}-Z_{Legislatove,k,r},{∆}_{k2,r}=Z_{Captiv,k,r}-Z_{STN,k,r},\;{∆}_{k3,r}=Z_{STN,k,r}-Z_{Legislative,k,r},$$

where $Z_{Captiv,k}$ represents the percentage in the corresponding working posture obtained by a given method, k=1,2,…,n determines the *k*-th measurement part of the run, *n* is the number of measurements stressed body parts, and *r* is the worker’s order, r=1,2,…,5. The difference represents how much the proportion in a given area increased (${∆}_{ki,r}>0$) or decreased (${∆}_{ki,r}<0$) for i=1,2,3.

## 3. Ergonomic Risk Assessment Applying Three Alternative Standards

The collected experimental data offered information about the time individual body segments spent in a specific working posture for five persons. Data were then evaluated by three risk assessment methods to classify the risk level for all working postures as acceptable (Green), conditionally acceptable (Orange), or unacceptable (Red). The next step was to determine the difference in results between three standards: the SK Legislation (in the graphs as L), the STN EN (in the graphs as S), and the Captiv system (in the graphs as C).

To determine the degree of physical load, we monitored the time of individual body segments spent in working postures expressed as a percentage during the work activity. Basic information about the tested workers is presented in Table 3.

**Figure 2:**
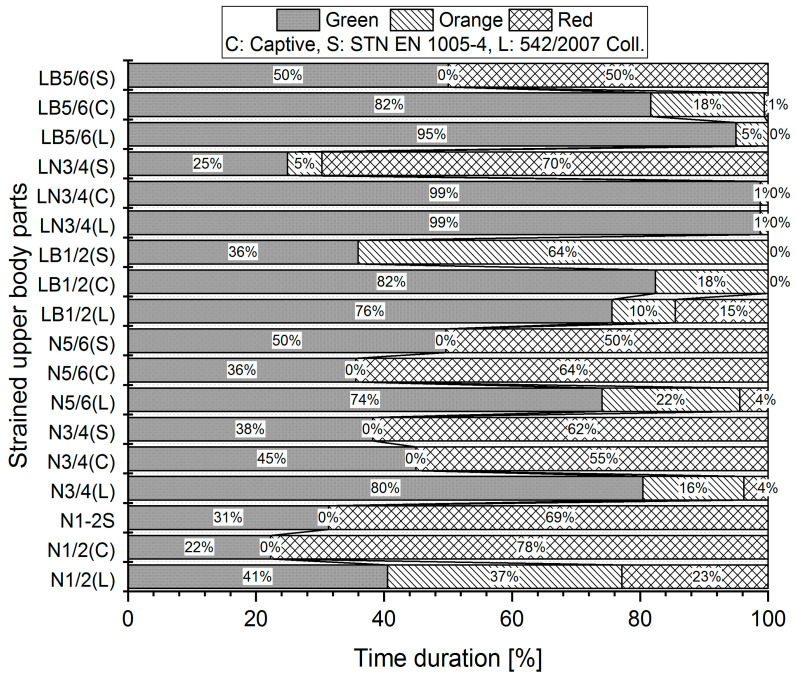
Percentage of body segments in planes of motion: neck, lower back—stressed body parts I (Worker 1). The evaluation was processed by three standard methods: L, S, C. The example shows the processed result of one worker.

**Figure 3:**
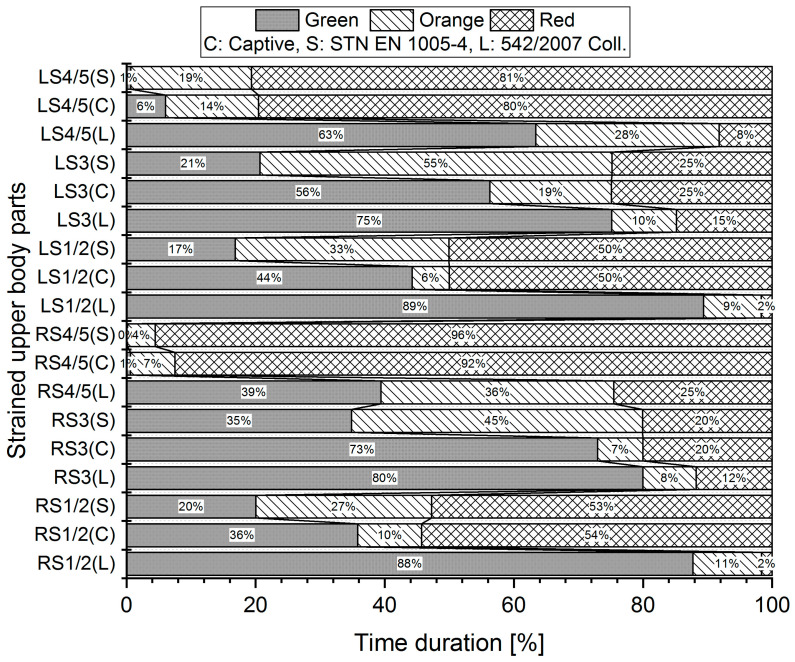
Percentage of body segments in planes of motion: left shoulder, right shoulder—stressed body parts II (Worker 1). The evaluation was processed by three methods: L, S, C. The example shows the processed results of one worker.

**Figure 4:**
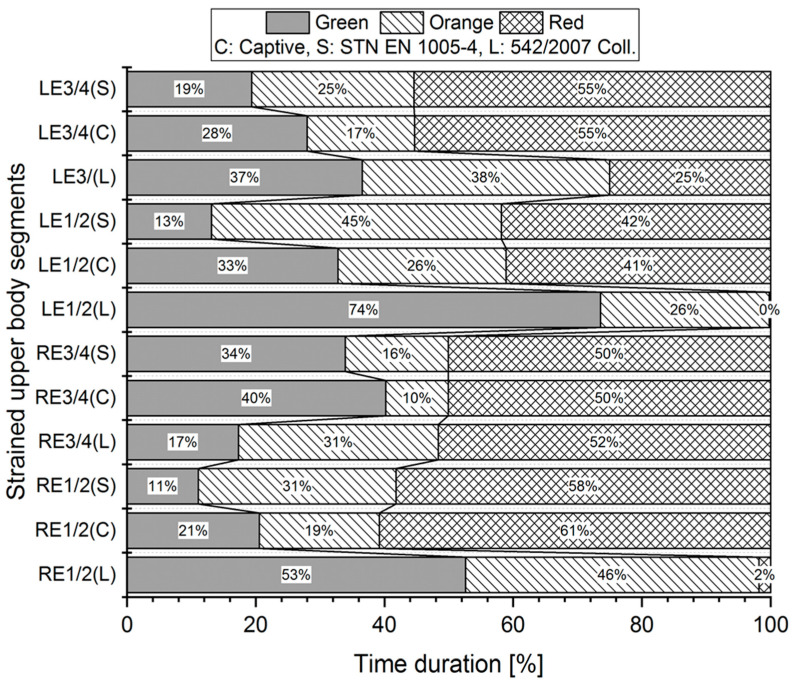
Percentage of body segments in planes of motion: left elbow, right elbow—stressed body parts III (Worker 1). The evaluation was processed by three methods: L, S, C. The example shows the processed results of one worker.

Measurements were made for each worker three times, and the mean angle data collected by the Captiv system were used in the evaluation by three different standards (L, S, C). For illustration, we provided a graphical representation of the measured values for evaluations according to the limit values of the Captiv, SK Legislation, and STN EN standards in the case of Worker 1. The graphs in Figure 2, Figure 3 and Figure 4 show the percentage of time spent in acceptable (G—Green area), conditionally acceptable (O—Orange area), and unacceptable work postures (R—Red area). 

The vertical axis is created by all measured body segments with their descriptions. LB5/6(S) means LB—lower back, and (S) means calculation by the SK Legislation standard. Each part of the body obtains a set of three values of a risk level calculated by the rules stated by different physical load assessments (L—SK Legislation; C—Captiv; S—STN EN).

For example, the analysis of the results (Figure 2) shows that for the stressed part LB5/6, when using the S method, the body segment in question is spending 50% of the work activity in acceptable work positions (Green area) and 50% of the work activity in unacceptable positions (Red area). Using method C, the body segment spends up to 82% of the work activity in acceptable working positions (Green area) and 18% of the work activity in conditionally acceptable working positions (Orange area). Similar results are obtained using the L standard, where the body segment spends up to 95% of the work activity in acceptable work positions (Green area) and 5% in conditionally acceptable work positions (Orange area).

For example, the analysis of the results (Figure 3) shows that for the stressed RS1/2, using the S method, the body segment in question spends only 20% of the work activity in acceptable work positions (Green area), 27% in conditionally acceptable work positions (Orange area), and up to 53% of the work activity in unacceptable positions (Red area). In the case of method C, the percentage redistribution of work activity is as follows: 36% of work activity in acceptable work positions (Green area), 10% in conditionally acceptable work positions (Orange area), and 54% of work activity in unacceptable positions (Red area). The results obtained with the L method are quite different: the body segment in question accounts for up to 88% of work activity in acceptable working positions (Green area), 11% in conditionally acceptable working positions (Orange area), and only 2% of work activity in unacceptable positions (Red area). Significant differences in the evaluation of the percentage of the given body segments are also shown in Figure 4.

**Figure 5:**
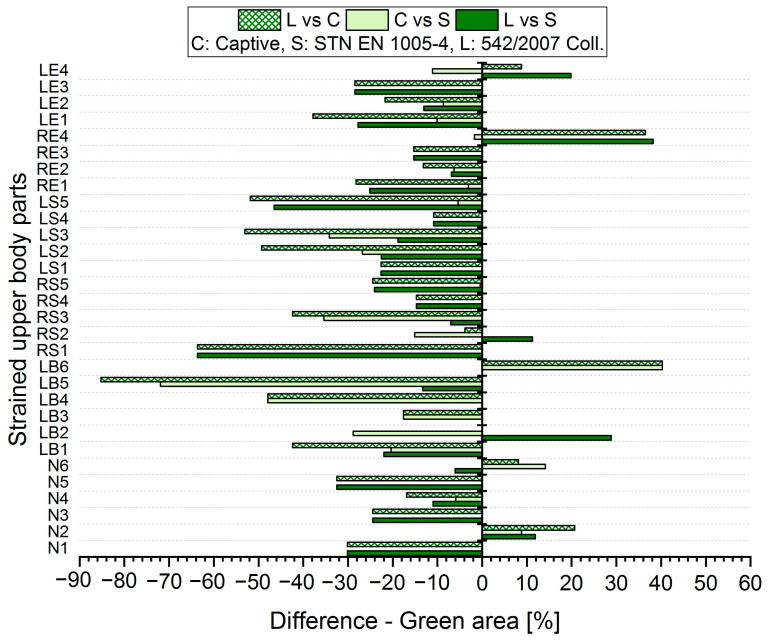
Differences in particular methods for the acceptable Green area (Worker 1).

**Figure 6:**
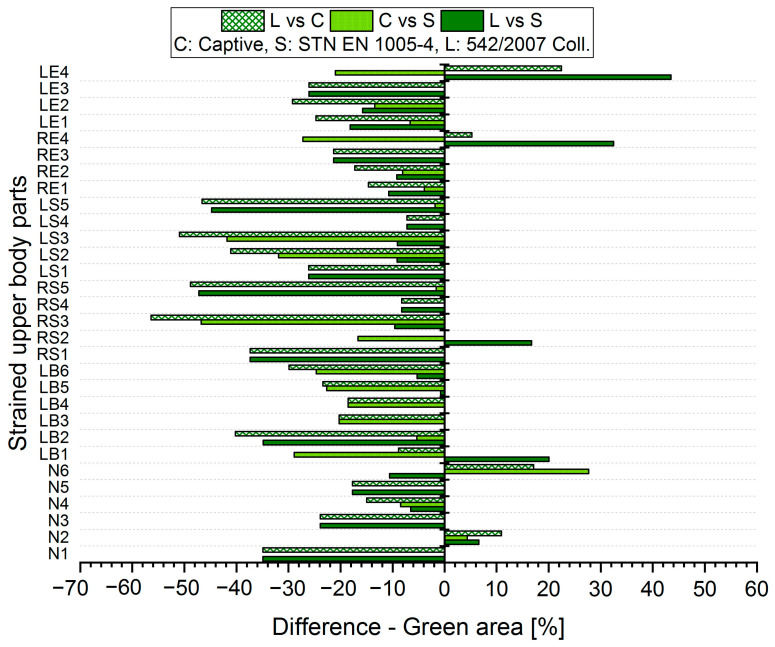
Differences in particular methods for the acceptable Green area (Worker 2).

**Figure 7:**
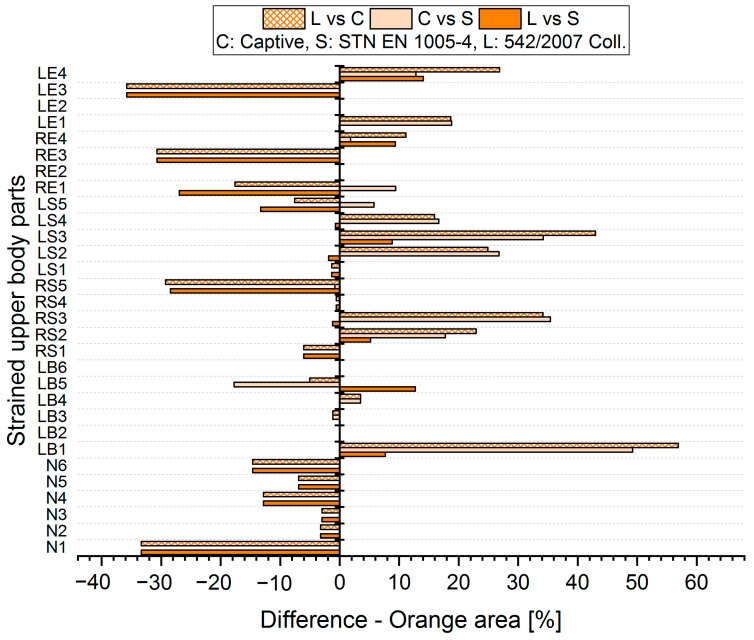
Differences in particular methods for the Orange area (Worker 1).

**Figure 8:**
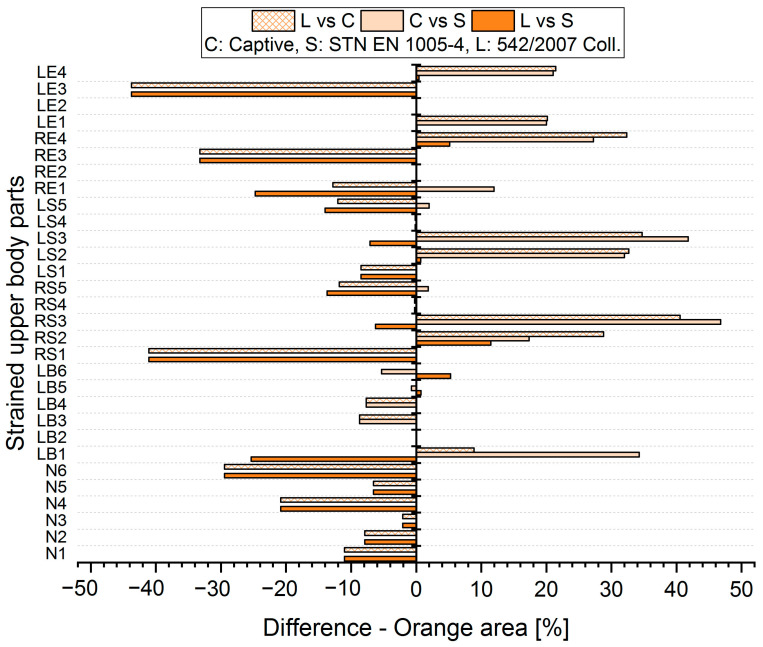
Differences in particular methods for the Orange area (Worker 2).

**Figure 9:**
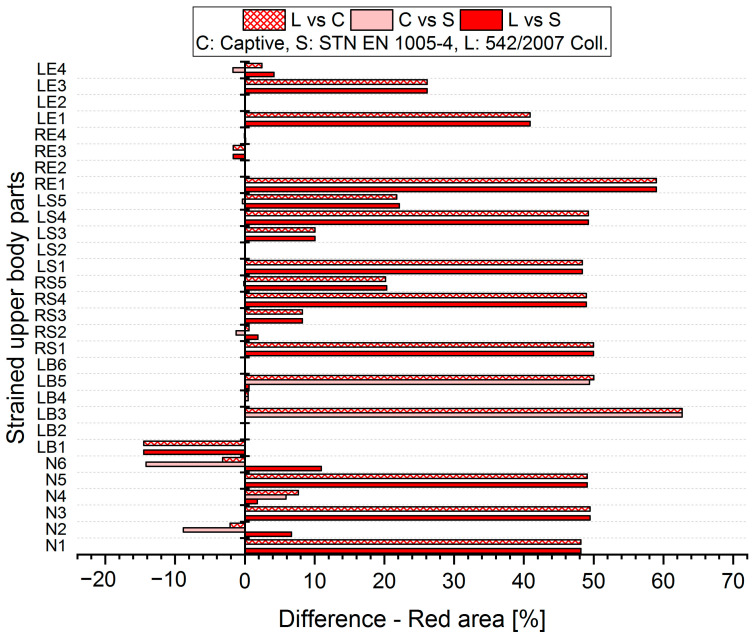
Differences in particular methods for the Red area (Worker 1).

**Figure 10:**
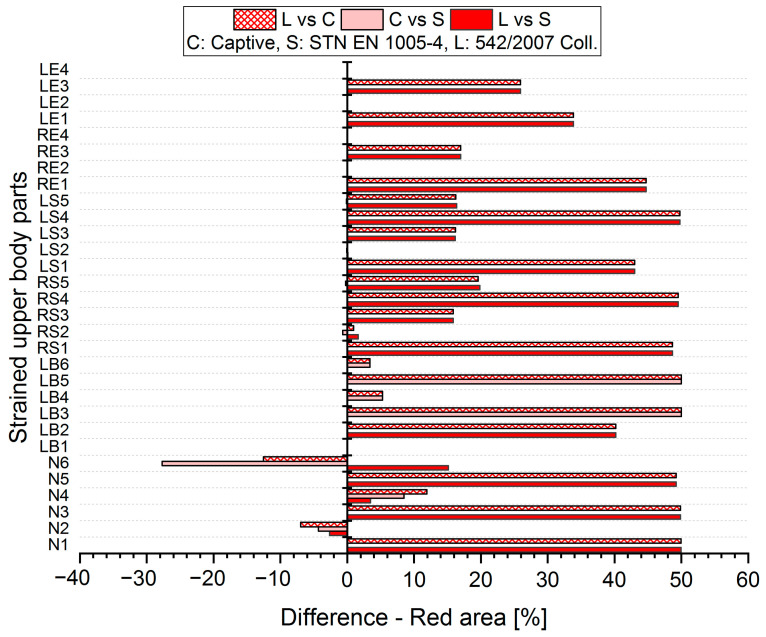
Differences in particular methods for the Red area (Worker 2).

The results analysis shows that the obtained values of the percentages of time spent in acceptable, conditionally acceptable, and unacceptable working postures differ in terms of the threshold values or assessment methods used. For each worker, for each body segment and in each working position (acceptable—Green area, conditionally acceptable—Orange area, unacceptable—Red area), we used Relation (1) to calculate the differences in the measured values, which were obtained by means of the S, C, and L assessment methods. Graphical representations of the differences for the first two workers are shown in Figure 5, Figure 6, Figure 7, Figure 8, Figure 9 and Figure 10.

The resulting values of the differences in the acceptable posture (Green area, Figure 5 and Figure 6) show that when comparing the SK Legislation and Captiv methods (L vs. C), the Captiv method significantly reduces the percentage in the acceptable posture in most of the stressed body parts (the differences are negative). The case is similar when comparing the SK Legislation and STN (L vs. S) or Captiv and STN (C vs. S) methods.

The resulting values of the differentials for the unacceptable position (Red area, Figure 9 and Figure 10) show that when comparing the SK Legislation and Captiv methods (L vs. C), the Captiv method significantly increases the percentage in the unacceptable position in most of the stressed body parts (the differentials are positive). Similar results are obtained when comparing the SK Legislation and STN (L vs. S) or Captiv and STN (C vs. S).

It shows that when comparing the SK Legislation and Captiv methods, the Captiv method significantly reduces the percentage in an acceptable position in most of the stressed body parts (${∆}_{k1}<0$). On the other hand, it significantly increases the percentage of time spent in the unacceptable position in most of the stressed body parts for each worker (${∆}_{k1}>0$). The comparison between the SK Legislation and STN EN or between the Captiv and STN EN methods is analogous. 

**Figure 11:**
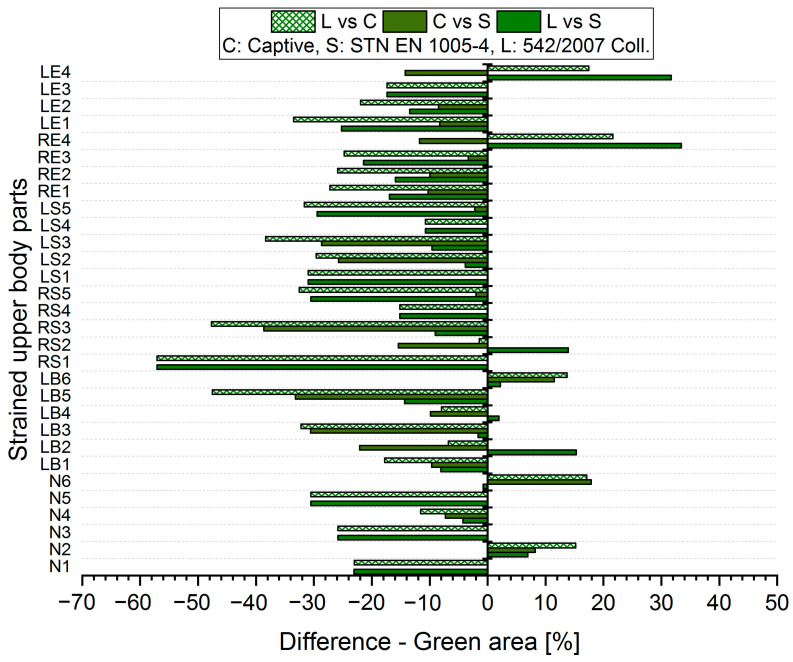
Resulting differences between assessment methods for the Green area.

**Figure 12:**
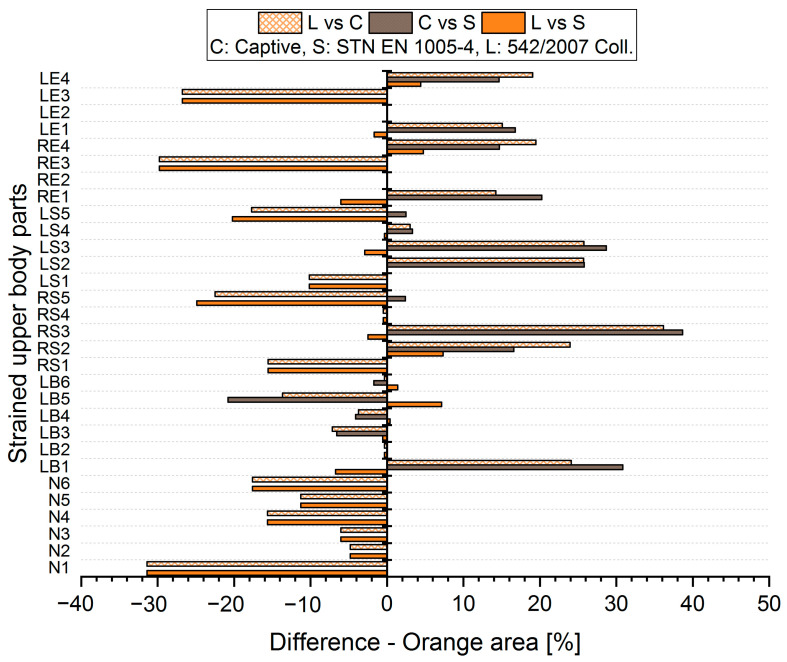
Resulting differences between assessment methods for the Orange area.

**Figure 13:**
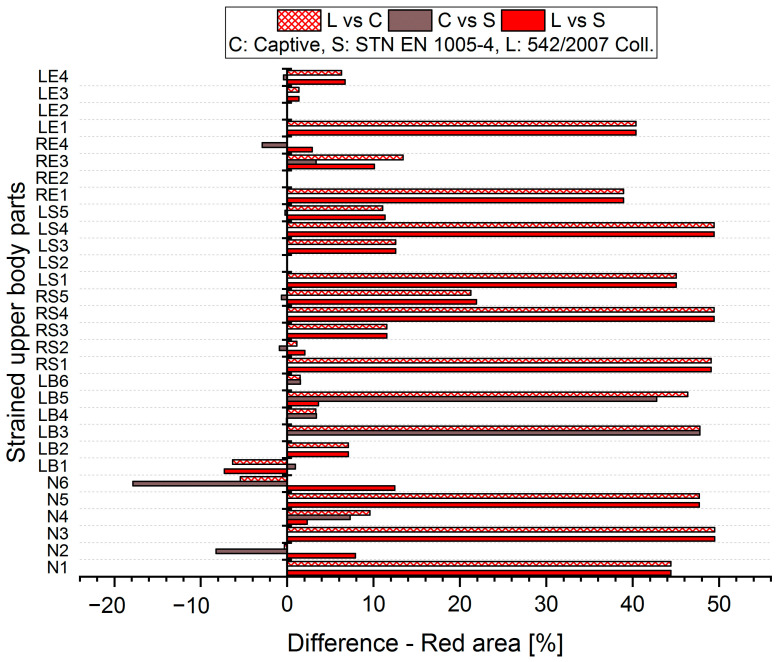
Resulting differences between assessment methods for the Red area.

The resulting mean differences ${∆}_{ki,r}$ at each location of the stressed body part in each position are graphically shown in Figure 11, Figure 12 and Figure 13.

In the next step, we test the equality of the means of the percentage of time the segments spend in each position out of the total work activity using a paired *t*-test. The null hypothesis is “the means of the different groups are the same” and the alternative hypothesis is “At least one sample mean is not equal to the others”. 

The prerequisite for using the paired *t*-test is to verify normality. The verification of the normality of the measured values is realized by the Shapiro–Wilk normality test. Since for each sample set, the *p*-value > α, we do not reject the null hypothesis of normality for each underlying set. In our case, all samples follow a normal distribution. The paired *t*-test results for Worker 1 in the Green work domain for SK Legislation vs. Captiv (L vs. C), Captiv vs. STN EN (C vs. S), and SK Legislation vs. STN EN (L vs. S) are shown in Table 4.

Because the *p*-value is below the significance level (*p*-value < α), we reject the null hypothesis of means equality. The results show that there are statistically significant differences between the values. The complete results of the 45 paired tests in all areas (Green, Orange, Red) for Worker 1 are in Table 5 and Table 6. If the *p*-value is less than the significance level, we can assume that the differences are significant (SD in the table).

As the results show, there are significant differences between the values measured by the L and C methods in all areas (Green, Orange, Red) and for all surveyed workers. There are significant differences (SDs) between the C and S methods in the Green and Orange areas. For the comparison between methods L and S, there are significant differences (SDs) in the Green and Red areas. There are no significant differences between methods C and S in the Red (N) area. In the case of the comparison between methods L and S, there are no significant differences in the Orange (N) area.

Table 7 shows the summary results. Worker 1 spent up to 67.46% of the total duration of work activity in the acceptable posture (Green area) and 20.86% in the conditionally acceptable posture (Orange area) according to the L method. Only 11.68% of the total duration of work activity was spent in the unacceptable posture (Red area). In terms of the C method, Worker 1 spent up to 45.44% of the total duration of work activity in the unacceptable posture. For the S assessment method, it was even up to 51.88% of the total duration of work activity. There are similar differences for the other workers.

The analysis of the results shows that workers were in an acceptable posture for approximately 63% of the total duration of the work activity in terms of the L method. Using the C method, approximately 44% of the total duration of work activity was spent in an acceptable posture. The least favorable rating was obtained for the S method, where workers spent only 27% of the total duration of work activity in an acceptable posture, which is 2.33 times less than the L method.

## 4. Discussion

A series of measurements were carried out on a sample of workers at a car headlight quality control assembly workplace using the Captiv wireless sensor system. Using the Captiv software, the measured data were processed. However, during the actual evaluation of the working postures and movements at work, obstacles were discovered with some differences in the applicable standards in the field of physical load assessment. The Decree 542/2007 Coll., which is in force in the territory of the Slovak Republic, and the STN EN 1005-4+A1 standard define the thresholds for risk positions at work with significant differences in several assessed body parts, which also affects the final evaluations of measurements. These standards were compared with the current French standards default in the Captiv system. We placed emphasis on determining whether the differences in values were statistically significant or whether the difference was negligible. We believe that our analysis of the existing standards and their possible modification is necessary to allow a more precise and valuable assessment of workload with reference to current standards in the future [9,34,35].

In our research, we did not attempt to decide on the correctness of any method. We intend to continue this research and extend the measurements with more workers and types of work in different professions. The main aim was to highlight the need for the refinement of existing methodologies, not only in terms of aligning national and European standards but also in terms of refining and unifying biomechanical models. These demands increased international research cooperation. Based on our observations, we found that the Captiv standards allow a wider analysis (whole body) for WMSD risk assessment, while the Slovak Legislation and the STN EN are more suitable and accurate for assessing the ergonomic risk of the upper limbs. This paper presents our approach towards an advanced monitoring and assessment system that enables ergonomic improvements, possibly lowering the high incidence of MSDs in industries in Slovakia, with our case study focusing on assembly operations in the automotive industry. We believe that the assessment of ergonomic risks for physically working persons using the wireless multisensory system Captiv, which captures working postures in real time, can help to improve the prevention of MSDs. This system is an advanced method for real-time measurements with monitoring instruments including wearable sensors for the measurement of exposure variables at work. The system is able to synchronize video records of workers’ motion with sensor data. During data processing, it evaluates ergonomic risks caused by the physical workload with respect to threshold values defined for different working positions and other physiological parameters as well. Such quantitative data sensing reduces the subjectivity and refines the ergonomic risk assessment.

We used the Captiv system in our study for physical load assessment at a car light quality control workplace, which is part of a machinery production line. Collected data were analyzed by three methods: the legislation Decree 542/2007 Coll. in Slovakia (SK Legislation), the standard STN EN 1005-4+A1 (STN EN), and the Captiv INRIS France-based standards. Both the SK Legislation and the STN EN assess the physical load at work based on the working postures of individual body segments. We found that the rules used in these three different standards do not use the same parameters for workload assessment. The threshold values determining postures in motion sequences as acceptable, unacceptable, or conditionally acceptable are different. Further, the motion is often related to the whole limb, not considering the movements and postures of particular smaller segments/joints. Therefore, we compared the results obtained from all three standards to identify how big those differences were. There are serious differences while evaluating the upper limbs. When evaluating the movements of the back, the SK Legislation coincides with the STN EN standard when defining unacceptable forward flexion and conditionally acceptable ranges of both forward flexion and extension. The STN EN matches with the Captiv system in defining the angle of lateral flexion. The limits used in the SK Legislation do not match the limits used in Captiv for static and dynamic types of work. The analysis of the assessment shows that the determination of the time the individual stressed parts spend in the different work areas (Green, Orange, Red) is also influenced by the choice of the assessment methodology (C, L, S). The analysis also discovered that workers were in an acceptable position for approximately 63% of the total duration of the work activity in terms of the L method, and approximately 44% of the total duration of work activity was in an acceptable posture using the C method. The least favorable rating was obtained for the S method, where workers spent only 27% of the total duration of work activity in an acceptable posture.

## 5. Conclusions

Our experiments confirmed that ergonomic assessment of physical worker activity with the wireless multisensory system Captiv, especially working posture capturing, can help to improve the quality and speed of analysis for the prevention of MSDs. 

On the other hand, the results from our analysis show some differences in methods between the three different standards used in our study for the ergonomic risk assessment of workers’ positions; they must be further analyzed and adapted to obtain a uniform approach. 

We think that the Captiv assessment system allows us to provide fast and precise analyses for workplaces with incidences of MSDs for the whole body. We recommend using the current Slovak Legislation and the new, adapted, and translated STN EN standards in the assessment of upper extremities and not for the assessment of lower extremities.

There is a need for uniform standards for the ergonomic risk assessment of body posture. In our further research, it will be important to analyze and unify threshold values for parameters to calculate the ergonomic risk level of individual body segments, including the tolerance borders, to guarantee a trustworthy assessment of workers’ physical loads. 

We have already started cooperation with industries focusing on safety improvement through the implementation of exoskeletons as supporting technical devices in strenuous work, within our ergonomic prevention project. To select the right exoskeleton, it is essential to evaluate the work process, the physical workload, and eventually the mental load that accompanies the particular work task. 

## Figures and Tables

**Table 1:** Comparison of posture limits for unacceptable and conditionally acceptable postures among STN EN 1005-4+A1, SK Legislation 542/2007 Coll. SR, and Captiv (FR legislation).

Segment	Risk Level	Type of Work	SK Legislation (Coll. SR 542/2007)	STN EN (STN EN 1005-4)	Captiv
Head and Neck	Unacceptable	Static	Forward flexion without support 25°	Forward flexion 40°	/
			Extension without support	Lateral flexion > 10°	
			Lateral flexion/Rotation > 15°	Rotation > 45°	
		Dynamic	Forward flexion > 25°, f ≥ 2/min	Forward flexion 40°, f ≥ 2/min.	Forward flexion ≥ 30°
			Lateral flexion/Rotation 15°, f ≥ 2/min	Lateral flexion > 10°, f ≥ 2/min.	Extension ≥ 20°
				Rotation > 45°, f ≥ 2/min.	Lateral flexion ≥ 20°
					Rotation ≥ 30°
	Conditionally acceptable	Static	Forward flexion 25–40° with back support	/	/
		Dynamic	Forward flexion 25–40°, f = 2/min	Forward flexion 40°, f < 2/min.	Forward flexion ≥ 15°
			Extension < 15°, f < 2/min	Lateral flexion > 10°, f < 2/min.	Extension ≥ 10°
			Lateral flexion < 15°, f < 2/min	Rotation > 45°, f < 2/min.	Lateral flexion ≥ 10°
			Rotation < 15°, f < 2/min		Rotation ≥ 15°
Back	Unacceptable	Static	Forward flexion > 60°	Forward flexion > 60°	/
			Extension without support/significant lateral flexion/rotation > 20°	Lateral flexion/Rotation > 10°	
		Dynamic	Forward flexion ≥ 60°, f ≥ 2/min	Forward flexion > 20°, f ≥ 2/min.	Forward flexion ≥ 45°
			Significant lateral flexion/rotation > 20°, f ≥ 2/min.	Extension, f ≥ 2/min.	Extension ≥ 20°
				Lateral flexion/Rotation > 10°, f ≥ 2/min.	Lateral flexion ≥ 20°
					Rotation ≥ 30°
	Conditionally acceptable	Static	Forward flexion without support 40–60°	Forward flexion with support 20–60°	/
			Extension with support	Extension with support	
			Lateral flexion/Rotation > 10° a < 20°		
		Dynamic	Forward flexion 60°, f = 2/min.	Forward flexion > 60°, f < 2/min.	Forward flexion ≥ 30°
			Extension, f < 2/min.	Extension, f < 2/min.	Extension ≥ 10°
			Lateral flexion right/left > 20°, f < 2/min.	Lateral flexion/Rotation > 10°, f < 2/min.	Lateral flexion ≥ 10°
					Rotation ≥ 15°
Upper limb	Unacceptable	Static	Shoulder flexion > 60°	Flexion > 60°	/
			Awkward positions	Extension	
				Abduction > 60°	
				Adduction	
		Dynamic	Shoulder flexion > 60°, f ≥ 2/min.	Flexion > 60°, f ≥ 2/min	Vertical rotation right/left ≥ 90°
			Shoulder extension, f ≥ 2/min.	Extension, f ≥ 2/min.	Horizontal rotation right/left −90°/30°
				Abduction > 60°, f ≥ 2/min.	Rotation internal/external −60°/45°
				Adduction, f ≥ 2/min.	
	Conditionally acceptable	Static	Shoulder flexion 40–60° without support	Flexion 20–60° with shoulder support	/
				Abduction 20–60° with shoulder support	
		Dynamic	Shoulder flexion 40–60°, f = 2/min.	Flexion > 60°, Extension, f < 2/min.	Vertical rotation right/left ≥ 60°
			Shoulder extension, f < 2/min.	Abduction > 60°, Adduction, f < 2/min	Horizontal rotation right/left −70°/10°
				Flexion 20–60°, f ≥ 2/min.	Rotation internal/external −40°/20°
				Abduction 20–60°, f ≥ 2/min.	
Lower limb	Unacceptable	Static	Extreme positions of knee/ankle	UNDEFINED	/
		Dynamic	Movements close to range of motion limits, f ≥ 2/min.		Rotation internal/external 30°/−20°
					Flexion/extension 100°/−20°
					Abduction/adduction 30°/−20°
	Conditionally acceptable	Static	/		/
		Dynamic	Movements close to range of motion limits, f < 2/min.		Rotation internal/external 10°/−10°
					Flexion/extension 70°/−10°
					Abduction/adduction 20°/−10°
Other body segments			Extreme positions, uncomfortable	Extreme positions, uncomfortable	-

**Table 2:** Measured body segments and their labels.

Joint	Movement	Label	Joint	Movement	Label
Neck	flexion	N1	Lower back	forward flexion	LB1
	extension	N2		extension	LB2
	lateral flexion right	N3		lateral flexion right	LB3
	lateral flexion left	N4		lateral flexion left	LB4
	rotation right	N5		rotation right	LB5
	rotation left	N6		rotation left	LB6
Right shoulder	rotation external	RS1	Left shoulder	rotation external	LS1
	rotation internal	RS2		rotation internal	LS2
	vertical rotation	RS3		vertical rotation	LS3
	horizontal rotation external/	RS4		horizontal rotation external	LS4
	horizontal rotation internal	RS5		horizontal rotation internal	LS5
Right elbow	flexion	RE1	Left elbow	flexion	LE1
	extension	RE2		extension	LE2
	rotation external	RE3		rotation external	LE3
	rotation internal	RE4		rotation internal	LE4

**Table 3:** Basic characteristics of workers.

Worker	Gender	Age	Weight [kg]	Height [m]	BMI
Worker 1	F	23	76	1.68	26.93
Worker 2	F	45	52	1.68	18.42
Worker 3	F	45	81	1.68	28.70
Worker 4	M	46	76	1.77	24.26
Worker 5	M	45	86	1.85	25.13

**Table 4:** Paired *t*-test result, Green area, Worker 1 (α = 0.05).

Results	SK Legislation/Captiv	Captiv/STN EN	SK Legislation/STN EN
*t*-test	4.160	6.847	3.371
*p*-value	0.0008	0.000005	0.0042
Conclusion	H0 rejected	H0 rejected	H0 rejected

**Table 5:** Complete test results—Worker 1, Worker 2, Worker 3 (α = 0.05).

Area	Worker 1			Worker 2			Worker 3		
	L vs. C	C vs. S	L vs. S	L vs. C	C vs. S	L vs. S	L vs. C	C vs. S	L vs. S
Green									
*p*-value	<0.001	<0.001	<0.001	<0.001	<0.001	<0.001	0.004	**0.080**	<0.001
Conclusion	SD	SD	SD	SD	SD	SD	SD	N	SD
Orange									
*p*-value	0.016	0.007	**0.659**	<0.001	0.006	**0.672**	0.014	**0.362**	**0.401**
Conclusion	SD	SD	N	SD	SD	N	SD	N	N
Red									
*p*-value	<0.001	**0.275**	<0.001	<0.001	**0.323**	<0.001	<0.001	**0.372**	<0.001
Conclusion	SD	N	SD	SD	N	SD	SD	N	SD

Note: SD—significant difference; N—insignificant difference; bold means maximal value.

**Table 6:** Complete test results—Worker 4, Worker 5 (α = 0.05).

Area	Worker 4			Worker 5		
	L vs. C	C vs. S	L vs. S	L vs. C	C vs. S	L vs. S
Green						
*p*-value	**0.060**	<0.001	<0.001	0.001	0.002	<0.001
Conclusion	N	SD	SD	SD	SD	SD
Orange						
*p*-value	<0.001	0.006	**0.469**	0.020	0.003	**0.798**
Conclusion	SD	SD	N	SD	SD	N
Red						
*p*-value	<0.001	**0.216**	<0.001	<0.001	**0.405**	<0.001
Conclusion	SD	N	SD	SD	N	SD

Note: SD—significant difference; N—insignificant difference; bold means maximal value.

**Table 7:** Percentage weight of total time workers spend in risk zones in their job.

Worker	Percentage Weight of Total Work Activity Workers Spend in Risk Zones in their Job [%]								
	Legislation			Captiv			STN EN		
	Green	Orange	Red	Green	Orange	Red	Green	Orange	Red
Worker 1	67.46	20.86	11.68	43.91	10.65	45.44	25.04	23.08	51.88
Worker 2	62.76	25.07	12.17	43.04	8.06	48.90	23.18	22.67	54.15
Worker 3	57.20	24.30	18.50	37.78	11.68	50.54	28.27	17.78	53.94
Worker 4	59.36	23.30	17.34	46.09	6.97	46.94	28.01	18.82	53.17
Worker 5	68.49	18.98	12.53	47.59	9.42	42.99	33.54	20.44	46.02
Average	63.05	22.50	17.92	43.68	9.36	46.96	27.61	20.56	51.83

## Data Availability

Data available on request due to restrictions, e.g., privacy or ethics. The data presented in this study are available upon request from the corresponding author. The data are not publicly available due to measures in the private industrial sector.
